# Detection of elevated right ventricular extracellular volume in pulmonary hypertension using Accelerated and Navigator-Gated Look-Locker Imaging for Cardiac T1 Estimation (ANGIE) cardiovascular magnetic resonance

**DOI:** 10.1186/s12968-015-0209-y

**Published:** 2015-12-21

**Authors:** Bhairav B. Mehta, Daniel A. Auger, Jorge A. Gonzalez, Virginia Workman, Xiao Chen, Kelvin Chow, Claire J. Stump, Sula Mazimba, Jamie L. W. Kennedy, Elizabeth Gay, Michael Salerno, Christopher M. Kramer, Frederick H. Epstein, Kenneth C. Bilchick

**Affiliations:** Department of Medicine, University of Virginia Health System, P.O. Box 800158, Charlottesville, VA 22908 USA; Department of Biomedical Engineering, University of Virginia Health System, Charlottesville, VA USA; Department of Radiology, University of Virginia Health System, Charlottesville, VA USA

**Keywords:** Magnetic resonance, Pulmonary hypertension, Fibrosis, T1 mapping, Extracellular volume fraction, Right ventricle

## Abstract

**Background:**

Assessment of diffuse right ventricular (RV) fibrosis is of particular interest in pulmonary hypertension (PH) and heart failure (HF). Current cardiovascular magnetic resonance (CMR) T1 mapping techniques such as Modified Look-Locker inversion recovery (MOLLI) imaging have limited resolution, but accelerated and navigator-gated Look-Locker imaging for cardiac T1 estimation (ANGIE) is a novel CMR sequence with spatial resolution suitable for T1 mapping of the RV. We tested the hypothesis that patients with PH would have significantly more RV fibrosis detected with MRI ANGIE compared with normal volunteers and patients having HF with reduced (LV) ejection fraction (HFrEF) without co-existing PH, independent of RV dilitation and dysfunction.

**Methods:**

Patients with World Health Organization group 1 or group 4 PH, patients with HFrEF without PH, and normal volunteers were recruited to undergo contrast-enhanced CMR. RV and LV extracellular volume fractions (RV-ECV and LV-ECV) were determined using pre-contrast and post-contrast T1 mapping using ANGIE (RV and LV) and MOLLI (LV only).

**Results:**

Thirty-two participants (53.1 % female, median age 52 years, IQR 26–65 years) were enrolled, including *n* = 12 with PH, *n* = 10 having HFrEF without co-existing PH, and *n* = 10 normal volunteers. ANGIE ECV imaging was of high quality, and ANGIE measurements of LV-ECV were highly correlated with those of MOLLI (*r* = 0.91; *p* < 0.001). The RV-ECV in PH patients was 27.2 % greater than the RV-ECV in normal volunteers (0.341 v. 0.268; *p* < 0.0001) and 18.9 % greater than the RV-ECV in HFrEF patients without PH (0.341 v. 0.287; *p* < 0.0001). RV-ECV was greater than LV-ECV in PH (RV-LV difference = 0.04), but RV-ECV was nearly equivalent to LV-ECV in normal volunteers (RV-LV difference = 0.002) (*p* < 0.0001 for RV-LV difference in PH versus normal volunteers). RV-ECV was linearly associated with both increasing RVEDVI (*p* = 0.049) and decreasing RVEF (*p* = 0.04) in a multivariable linear model, but PH was still associated with greater RV-ECV even after adjustment for RVEDVI and RVEF.

**Conclusions:**

Pre- and post-contrast ANGIE imaging provides high-resolution ECV determination for the RV. PH is independently associated with increased RV-ECV even after adjustment for RV dilatation and dysfunction, consistent with an independent effect of PH on fibrosis. ANGIE RV imaging merits further clinical evaluation in PH.

## Background

Right ventricular (RV) dysfunction in pulmonary hypertension (PH) is not only an indicator of severity of disease but also a cause of heart failure (HF) [1, 2] and the most important predictor of survival [3–6]. RV pathophysiology in PH and HF involves complex interactions among myocardial injury, altered gene expression, ventricular remodeling, the renin-angiotensin-aldosterone system, natriuretic peptides, the endothelin system, and various cytokines, leading to fibrosis, cell death, myocardial dysfunction, and reduced systemic perfusion [7–9]. Left ventricular (LV) fibrosis is of considerable interest and readily evaluated in hypertension and other cardiovascular diseases [10], while RV fibrosis is more difficult to assess than LV fibrosis because of the specific attributes of RV anatomy, including the thin RV free wall, differences in structure between the septum and free wall, and the complex 3D structure including an outflow tract. Although echocardiography provides some information on RV structure and function [11], cardiovascular magnetic resonance (CMR) is generally considered the gold standard in this regard, even though CMR assessment of fibrosis in PH has been limited to evaluation of scar by late gadolinium enhancement (LGE) at LV septal insertion sites [12, 13]. Consequently, the strength of CMR for the RV has traditionally been high-definition cine imaging with a steady-state free precession protocol rather than contrast-enhanced fibrosis imaging.

There is a growing literature for T1 mapping of the LV, and the myocardial extracellular volume (ECV) fraction determined with this technique has been shown to correlate with histologic fibrosis [14–22]; however, current T1 mapping methods have insufficient spatial resolution for effective RV fibrosis imaging [14]. For example, limitations of common T1 mapping methods such as modified Look-Locker inversion recovery imaging (MOLLI) [14] include suboptimal spatial resolution and the need for breath holding during scanning. In response, our group has developed a method for high quality CMR T1 mapping of the RV called accelerated and navigator-gated Look-Locker imaging for cardiac T1 estimation (ANGIE) [20], which employs navigator gating and compressed sensing to provide a 4-fold improvement in spatial resolution compared with MOLLI without the need for a breath hold.

Considering that we have previously demonstrated the accuracy of ANGIE for native T1 mapping of the LV and RV [20], the aim of the present study was to demonstrate the feasibility and effectiveness of contrast-enhanced ANGIE imaging for determination of RV-ECV and LV-ECV in a cross-sectional study composed of a) patients with World Health Organization (WHO) groups 1 or 4 PH (without LV systolic dysfunction), b) patients having HF with reduced (LV) ejection fraction (LVEF) (HFrEF) without co-existing PH, and c) normal volunteers. Because the RV in PH is compromised by high pressures, which lead to chamber dilitation, dysfunction, and fibrosis, we hypothesized that patients with PH would have significantly more RV fibrosis detected with ANGIE compared with normal volunteers and patients having HF with reduced (LV) ejection fraction without co-existing PH, independent of RV dilitation and dysfunction.

## Methods

### Patient cohort

All participants provided written informed consent, and the study was conducted according to standard ethical principles, as documented in the study protocol approved by our Institutional Review Board for Human Subjects Research. Patients were recruited to undergo CMR with a gadolinium-based contrast agent. The study population was divided into three groups of subjects. The PH cohort (*n* = 12) consisted of patients diagnosed with PH classified as WHO group 1 (pulmonary arterial hypertension) or 4 (chronic thromboembolic PH). WHO groups 1 and 4 were chosen for the PH cohort rather than WHO groups 2 (PH due to left heart diseases), 3 (PH due to lung diseases and/or hypoxemia), or 5 (PH with unclear multifactorial mechanisms) in order to provide a PH cohort with preserved LV systolic function without extensive comorbid cardiopulmonary disease. The second group consisted of patients with HFrEF without PH (*n* = 10). The third group (*n* = 10) consisted of healthy volunteers who served as the control group for this study. Patients were excluded from the study if they had standard contraindications for gadolinium-enhanced CMR. All patients were required to have a glomerular filtration rate (GFR) of at least 45 cc/minute/1.73 m^2^ (calculated based on a serum creatinine drawn within 30 days of the CMR study using the Modification of Diet in Renal Disease [MDRD] equation). Although we did not enroll patients on intravenous prostacyclin, these patients could be included in future studies, but would require nursing assistance with the tubing extension to maintain a connection with the pump during the scan.

### CMR protocol and image reconstruction

All CMR studies were performed using a 1.5 T MRI scanner (Avanto, Siemens, Erlangen, Germany). Peripheral intravenous access was obtained, and the hematocrit was determined from a peripheral blood draw for the calculation of ECV, as described in more detail below. After electrocardiography leads were placed, each patient was positioned in the magnet. A 5-channel phased-array radiofrequency coil was used for signal reception. With respect to the CMR imaging protocol for this study, a standard localizer was first performed to identify the cardiac short- and long-axis planes. Steady-state free precession cine images were then obtained for assessment of the RV end-diastolic volume index (RVEDVI), the LV end-diastolic volume index (LVEDVI), RV end-systolic volume index (RVESVI), LV end-systolic volume index (LVESVI), RVEF, and LVEF. Specific parameters included: TR = 2.7 ms, TE = 1.3 ms, flip angle = 70°, FOV = 300–350 mm, and in-plane spatial resolution = 1.8 × 1.4 mm^2^. The entire heart was covered using a stack of short-axis slices with a thickness of 8 mm and an inter-slice gap of 2 mm. Three long-axis slices were also acquired in two-chamber, three-chamber, and four-chamber views.

Next, pre-contrast ANGIE T1 mapping was performed. For ANGIE, two mid-ventricular short-axis slices were imaged at end systole to maximize RV wall thickness and separation of the RV wall from the liver and the chest wall during imaging. The mid-ventricular short-axis slices were aligned perpendicular to the major-axis of the RV and positioned in a region with minimal variation along the through plane direction in order to minimize the partial volume effects. The ANGIE protocol utilized repetitions of an inversion pulse followed by four consecutive ECG-triggered data acquisitions and a recovery period of two R-R intervals, as well as the following sequence parameters: TR = 3.2 ms, TE = 1.6 ms, flip angle = 35°, FOV = 270–340 mm, matrix size = 224 × 224, in-plane resolution = 1.2–1.4 × 1.2–1.4 mm^2^, slice thickness = 4 mm, acquisition window duration = 102 ms per heartbeat, phase encodes per heartbeat = 32, navigator acceptance window = ± 3 mm, initial inversion time = 160 ms, inversion time increment = 80 ms, and acceleration rate = 2. For all ANGIE scans, the raw k-space data were exported from the scanner, and image reconstruction was performed offline on a personal computer as previously described [20].

Following pre-contrast ANGIE, gadolinium-DTPA (Magnevist, Bayer Healthcare) was injected intravenously with a dose of 0.15 mmol/kg. LGE CMR was performed 10 min following gadolinium-DTPA injection at slice locations matching those acquired using cine CMR. A phase sensitive inversion recovery sequence was used with the inversion time set to null normal appearing myocardium. Specific sequence parameters included TR = 3.2 ms, TE = 1.6 ms, flip angle = 25°, FOV = 300–340 mm, resolution = 1.8 × 1.3 mm^2^, and slice thickness = 4 mm. The image acquisition was timed to occur at end systole for alignment with T1 mapping acquisitions. Post-contrast ANGIE T1 mapping acquisitions were performed at 20 min and 30 min after contrast administration using the same parameters and slice locations as the pre-contrast ANGIE T1 mapping acquisitions.

**Fig. 1:**
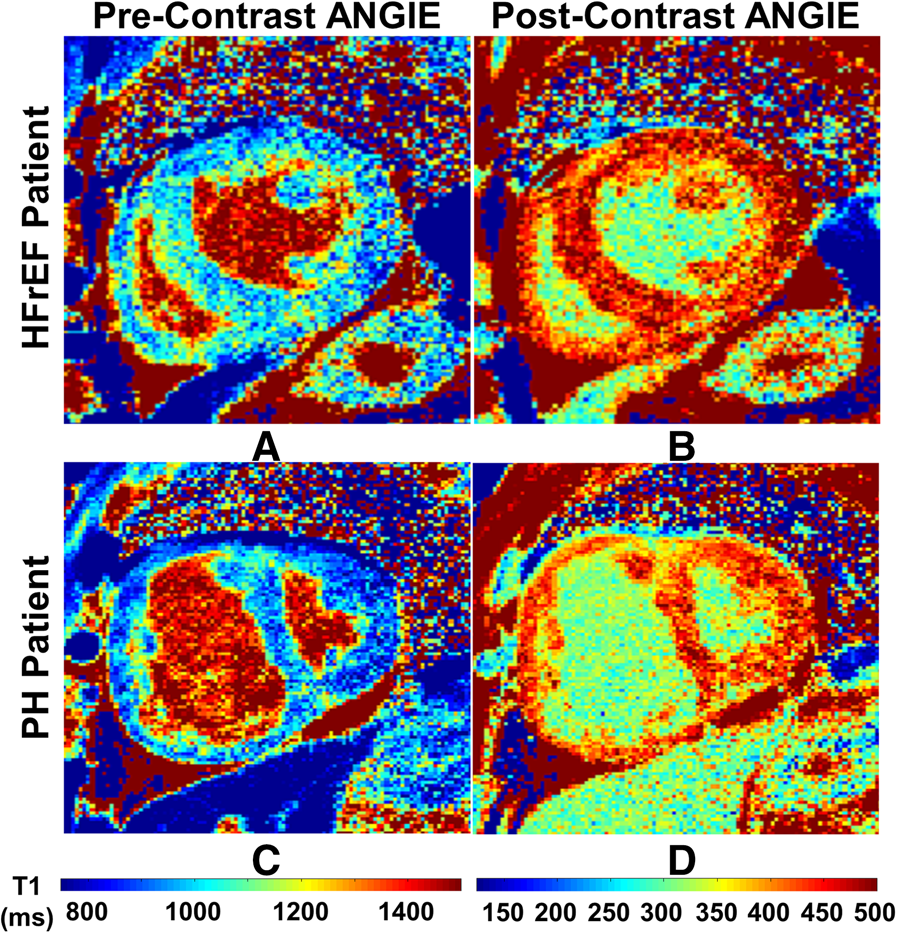
Examples of ANGIE T1 maps for PH and HFrEF. ANGIE T1 maps before and after injection of gadolinium in patients with heart failure with reduced ejection fraction (HFrEF) (**a**, **b**) and pulmonary hypertension (PH) (**c**, **d**) show excellent definition of the right ventricular (RV) free wall

Modified Look-Locker inversion recovery (MOLLI) T1 mapping scans were also performed both pre- and post-contrast in PH patients for validation of ANGIE LV-ECV measurements. For both pre- and post-contrast imaging, we used a shorter variant of MOLLI (MOLLI 5-(R4)-3) comprised of two inversion recovery based Look-Locker experiments [23]. The shorter MOLLI sequence acquired 5 images after the first inversion, used a four heart-beat pause, and then acquired three images after the second inversion. The slice locations matched those of the ANGIE acquisition, and the image acquisition was timed to occur at end systole for alignment with the ANGIE T1 maps. Specific imaging parameters for MOLLI were TR = 2.7 ms, TE = 1.16 ms, FOV = 300–310 mm, matrix size = 192 × 154, pixel size = 1.6–1.7 × 1.9–2.1 mm^2^, flip angle = 35°, slice thickness = 4 mm, acquisition window duration = 234 ms per heartbeat, initial TI = 100 ms, and TI increment = 80 ms. Only LV-ECV was determined with MOLLI because MOLLI did not provide sufficient resolution for a meaningful determination of RV-ECV. In contrast, ANGIE provided sufficient resolution for the RV [Fig. 1].

### CMR data analysis and ECV determination

Myocardial borders were manually delineated on steady state free precession cine images using Argus software (Siemens Medical Solutions, Munich, Germany) for determination of RVEDVI, LVEDVI, RVEF, and LVEF. All other image analysis was performed using custom software developed in MATLAB (The Mathworks, Inc., Natick, Massachusetts).

For ANGIE and MOLLI, T1 maps were computed as described previously [20]. Manual contours for the RV and LV were drawn in a conservative manner on images at one inversion time to exclude trabeculations. Non-scar regions of myocardium were identified for analysis of ECV by the absence of enhancement in the corresponding LGE image. The myocardial partition coefficient for gadolinium-DTPA (*λ*_*Gd*_) was computed from the myocardial and the LV blood T1 estimates using the standard equation [23–25]:

$$ {\lambda}_{Gd}=\frac{Gd\kern0.28em  concentration\kern0.28em  in\kern0.28em  myocardium}{Gd\kern0.28em  concentration\kern0.28em  in\kern0.28em  blood}=\frac{1/{T}_{1\kern0.28em myo\kern0.28em  with\kern0.24em Gd}-1/{T}_{1\kern0.28em myo\kern0.28em  with out\kern0.28em Gd}}{1/{T}_{1\kern0.28em  blood\kern0.28em  with\kern0.24em Gd}-1/{T}_{1\kern0.28em  blood\kern0.28em  with out\kern0.28em Gd}} $$

Myocardial ECV was calculated from *λ*_*Gd*_ and the blood hematocrit using the established relationship: *ECV* = *λ*_*Gd*_(1 − *hematocrit*) [23–25].

### Assessment of PH with echocardiography and right heart catheterization

Evaluation for the presence of PH in both groups of patients was assessed based on right heart catheterization and echocardiography results. The result obtained from the right heart catheterization was considered the gold standard. RV systolic pressures by echocardiography were assessed based on the peak tricuspid regurgitation jet velocity and right atrial pressure using the standard calculation based on the simplified Bernoulli equation.

### Statistical analysis

All statistical analyses were performed using SAS 9.4 (Carey, North Carolina) and SigmaPlot 12.5 (Systat Software, Inc., San Jose, California). Continuous variables were described using the median and interquartile range. The distribution of the continuous variables between groups was assessed using the Shapiro-Wilk test for normality. For selected variables with a confirmed normal distribution, the mean was also used. Comparisons of continuous variables between groups were performed using the Kruskal-Wallis test. Categorical values were described using frequencies and percentages in the different groups, and comparisons between groups were performed using Fisher’s exact test. Correlations between continuous variables were assessed using Pearson’s correlation coefficient after confirmation of normality and evaluated with scatter plots. Multivariable linear regression was used to assess the complex relationship between RV-ECV, RVEF, and RVEDVI, and the underlying clinical condition.

Interobserver variability was assessed for RV-ECV, pre-contrast T1, and post-contrast T1 in 10 randomly selected participants. The intraclass correlation coefficient (ICC) is reported as the primary measure of interobserver variability. 

## Results

### Patient cohort and baseline CMR parameters

Thirty-two participants were enrolled in the study (53.1 % female, median age 52 years old, IQR 26–65 years old). Patient characteristics for the entire cohort are described in Table 1. Of note, 75 % of PH patients were on advanced therapies for PH, including 50 % on an endothelin receptor antagonist (ERA), 58.3 % on sildenafil, and 16.7 % on a prostacyclin analog.

**Table 1:** Baseline characteristics

	PH (*N* = 12)	HFrEF (*N* = 10)	Normal (*N* = 10)	*p*-value
Age (years [IQR])	64.5 (47.5–70.5)	60.0 (53.0–65.0)	24.4 (21.7–26.3	<0.001
Gender (% female)	8 (66.7)	1 (10.0)	8 (80.0)	0.004
BMI (kg/m2 [IQR])	25.4 (23.0–29.3)	29.2 (26.0–32.7)	24.1 (21.2–26.3)	0.47
Ischemic HD (%)	1 (8.3)	8 (80.0)	0 (0.0)	<0.0001
Congenital HD (%)	1^a^ (8.3)	1 (10.0)	0 (0.0)	1.0
DM (%)	2 (16.7)	3 (30.0)	0 (0.0)	0.23
OSA (%)	3 (25.0)	2 (20.0)	0 (0.0)	0.33
HIV (%)	2 (16.7)	0 (0.0)	0 (0.0)	0.31
CTEPH (%)	2 (16.7)	0 (0.0)	0 (0.0)	0.31
Medication-ERA	6 (50.0)	0 (0.0)	0 (0.0)	0.002
Medication-sildenafil	7 (58.3)	0 (0.0)	0 (0.0)	0.0003
Medication-PA	2 (16.7)	0 (0.0)	0 (0.0)	0.31

Continuous variable are reported as median (IQR)

*CTEPH* chronic thromboembolic pulmonary hypertension, *DM* diabetes mellitus, *ERA* endothelin receptor antagonist, *HD* heart disease, *HIV* human immunodeficiency virus, *OSA* obstructive sleep apnea, *PA* prostacyclin analogue, *TE* thromboembolism

^a^This patient has severe pulmonary hypertension resulting from late repair of a ventricular septal defect

CMR parameters for PH patients, HFrEF patients, and healthy volunteers are shown in Table 2, which shows that RVEF was decreased, and both RVEDVI and RVESVI were increased in PH compared with HFrEF and normal volunteers (*p* < 0.05 for all). Specifically, RVEF was most significantly depressed in the PH group (34.0 % [IQR 30.5–42.5 %]), mild-moderately depressed in the HFrEF group (41.0 % [IQR 38.0–48.0 %], and normal in volunteers (55.5 % [IQR 52.0–58.2 %]). In HFrEF patients, LVEF was decreased (*p* < 0.0001), and LVEDVI (*p* = 0.0001), LVESVI (*p* = 0.001), and LVMI (*p* = 0.008) were increased compared with PH patients and normal volunteers.

Right heart catheterization was used for the diagnosis of PH. Patients with HFrEF had no evidence of PH by Doppler echocardiography. RV systolic pressure was twofold higher in the PH cohort compared with the HFrEF cohort by Doppler echocardiography (52.3 mm Hg versus 26.8 mm Hg; *p* = 0.01).

**Table 2:** CMR parameters

	PH (*N* = 12)	HFrEF (*N* = 10)	Normal (*N* = 10)	*p* value
RVEF (%)	34.0 (30.5–42.5)	41.0 (38.0–48.0)	55.5 (52.0–58.0)	0.001
RVEDVI (ml/m^2^)	87.9 (75.0–119.2)	59.0 (50.4–67.9)	60.8 (59.0-70.1)	0.02
RVESVI (ml/m^2^)	59.1 (47.7–80.9)	35.0 (23.0–37.4)	29.8 (26.8-32.4)	0.007
RV T1 pre-contrast (ms)	1056 (1021–1081)	1003 (922–1029)	974 (927–996)	0.005
RV T1 post-contrast (ms)	482 (428–509)	480 (449–506)	514 (503–538)	0.05
RV-ECV	0.343 (0.331-0.352)	0.294 (0.272-0.301)	0.270 (0.251-0.281)	<0.0001
LVEF (%)	55.0 (50.0–58.5)	28.0 (19.0–31.0)	61.2 (59.7–62.9)	<0.0001
LVEDVI (ml/m^2^)	58.2 (43.1–66.1)	109.0 (102.9–124.8)	84.3 (80.3–90.3)	0.0001
LVESVI (ml/m^2^)	28.2 (21.2–41.6)	62.3 (45.5–79.6)	32.7 (29.9–37.4)	0.001
LVMI (g/m^2^)	36.9 (32.7–41.0)	61.1 (50.7–70.5)	32.3 (30.1-35.8)	0.008
LV T1 pre-contrast (ms)	988 (963–1006)	969 (921–988)	971 (948–986)	0.40
LV T1 post-contrast (ms)	495 (438–542)	480 (454–505)	519 (491–546)	0.28
LV-ECV	0.305 (0.295-0.308)	0.276 (0.258-0.290)	0.271 (0.251-0.279)	0.002

Results are shown as the median (IQR)

*ECV* extracellular volume fraction, *LV* left ventricle, *LVEDVI* left ventricular end-diastolic volume index, *LVEF* left ventricular ejection fraction, *LVESVI* left ventricular end-systolic volume index, *RV* right ventricle, *RVEDVI* right ventricular end-diastolic volume index, *RVEF* right ventricular ejection fraction, *RVESVI* right ventricular end-systolic volume index

### ECV mapping results

**Fig. 2:**
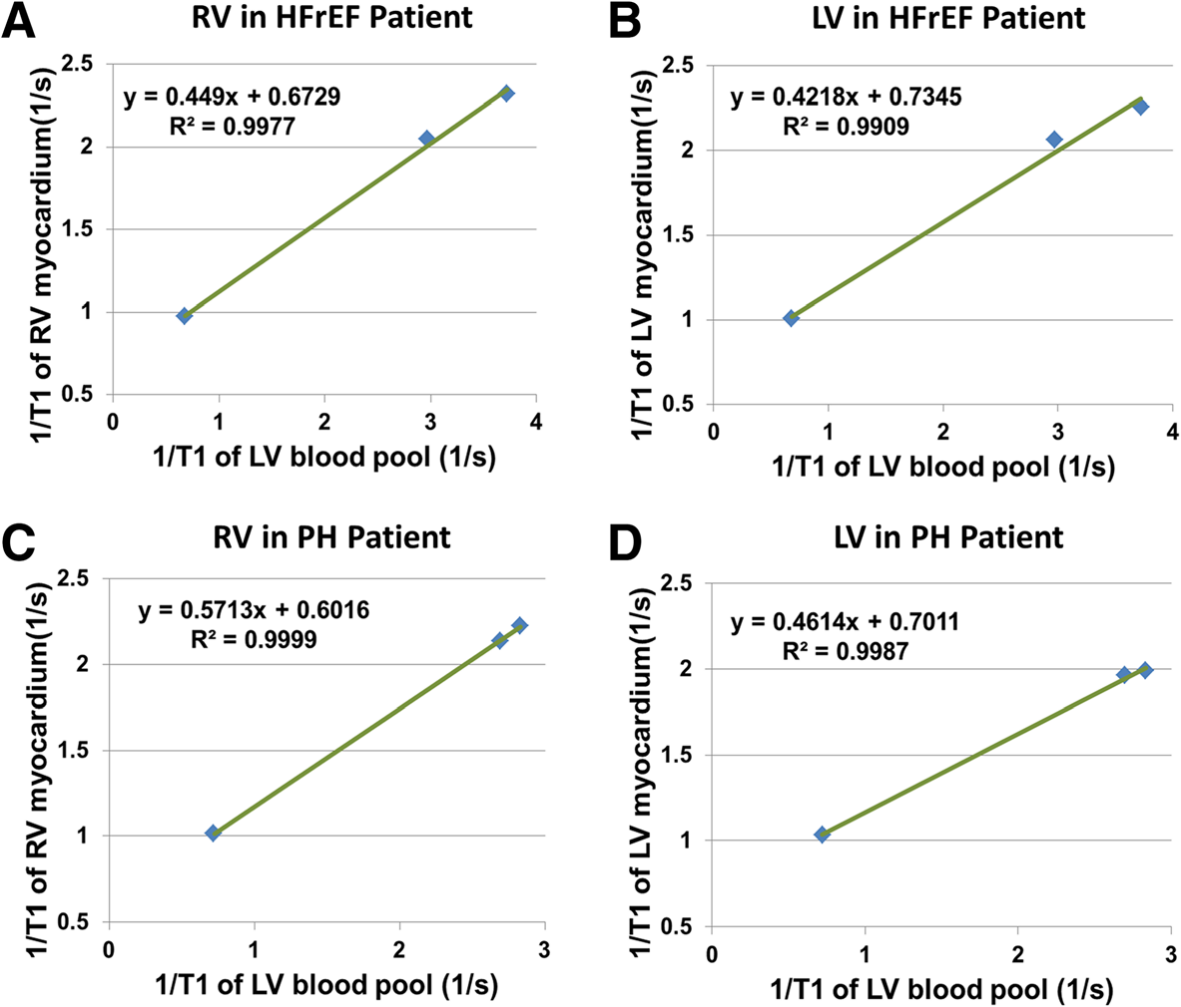
Myocardial partition coefficients for gadolinium-DTPA (λ_Gadolinium_) in patients with PH and HFrEF. Results for the right ventricular (RV) and left ventricular (LV) λ_Gadolinium_ are shown for patients with heart failure with reduced ejection fraction (HFrEF) (**a**, **b**) and pulmonary hypertension (PH) (**c**, **d**). λ_Gadolinium_ is estimated as the slope of the linear fit of 1/(myocardial T1) vs 1/ (LV blood pool T1) measured at various time points pre- and post-injection of gadolinium-DTPA

Examples of high-resolution pre- and post-contrast ANGIE T1 maps of the LV and RV acquired from HFrEF (a-b) and PH (c-d) patients are shown in Fig. 1. These examples illustrate the high quality of ANGIE T1 maps for this cohort of patients. The ANGIE T1 maps in both patients show good definition of not only the LV but also the RV. Figure 2 illustrates determination of the RV (A and C) and LV (B and D) myocardial partition coefficients for gadolinium-DTPA (*λ*_*Gd*_) using T1 estimates of the myocardium and LV blood pool.

### Correlation of ANGIE and MOLLI LV- ECV assessments

**Fig. 3:**
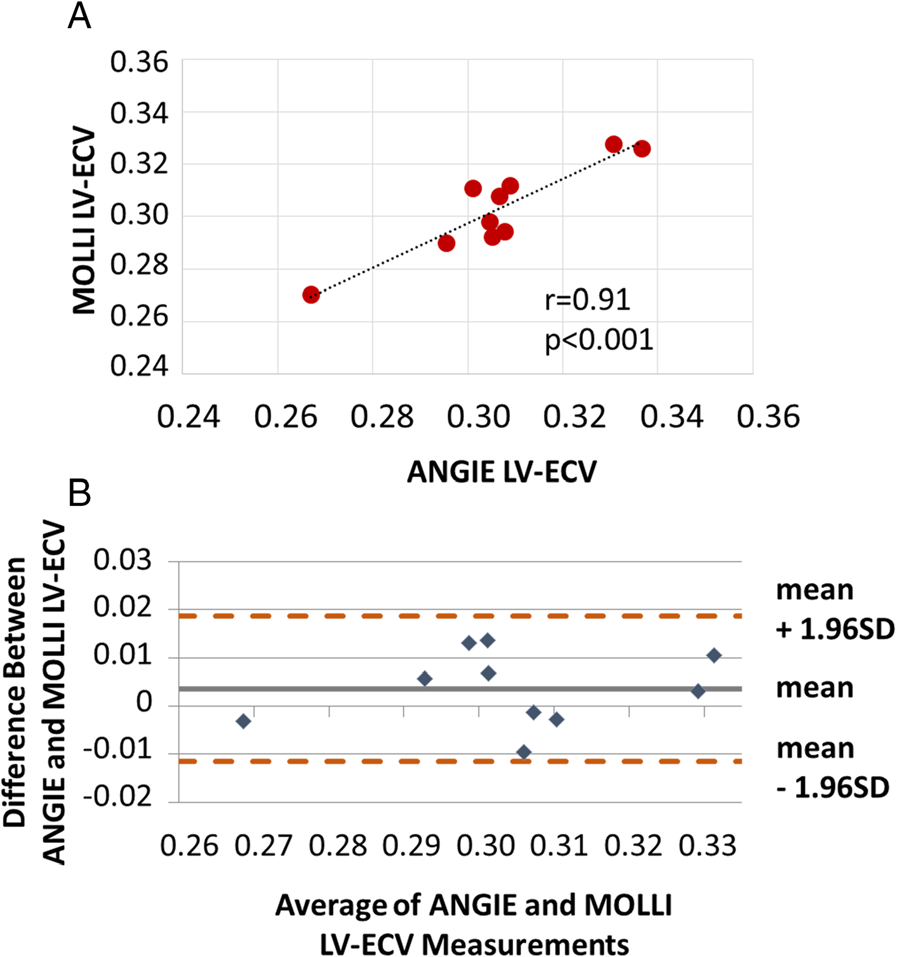
Validation of the accuracy of ECV measurements using ANGIE T1 mapping. **a** This panel shows the correlation plot comparing left ventricular extracellular volume (LV-ECV) in pulmonary hypertension (PH) obtained with accelerated and navigator-gated Look-Locker imaging for cardiac T1 estimation (ANGIE) versus the modified Look-Locker inversion recovery (MOLLI) imaging. The ANGIE measurements of LV-ECV (in regions excluding scar on late gadolinium enhancement) in PH patients were in close agreement with those of MOLLI (*r* = 0.91; *p* < 0.001), confirming the accuracy of ECV measurements performed using ANGIE. **b** The corresponding Bland-Altman plot is shown. SD = standard deviation

MOLLI acquisitions were obtained in addition to ANGIE in patients with PH (*n* = 12). The distribution of LV-ECV in these patients was determined to be normal. As shown in Fig. 3, the ANGIE measurements of LV-ECV in PH patients were highly correlated with MOLLI measurements (*r* = 0.91; *p* < 0.001), which supports the accuracy of myocardial ECV measurement using ANGIE T1 mapping.

### Comparison of RV-ECV in PH, HFrEF, and normal volunteers

**Fig. 4:**
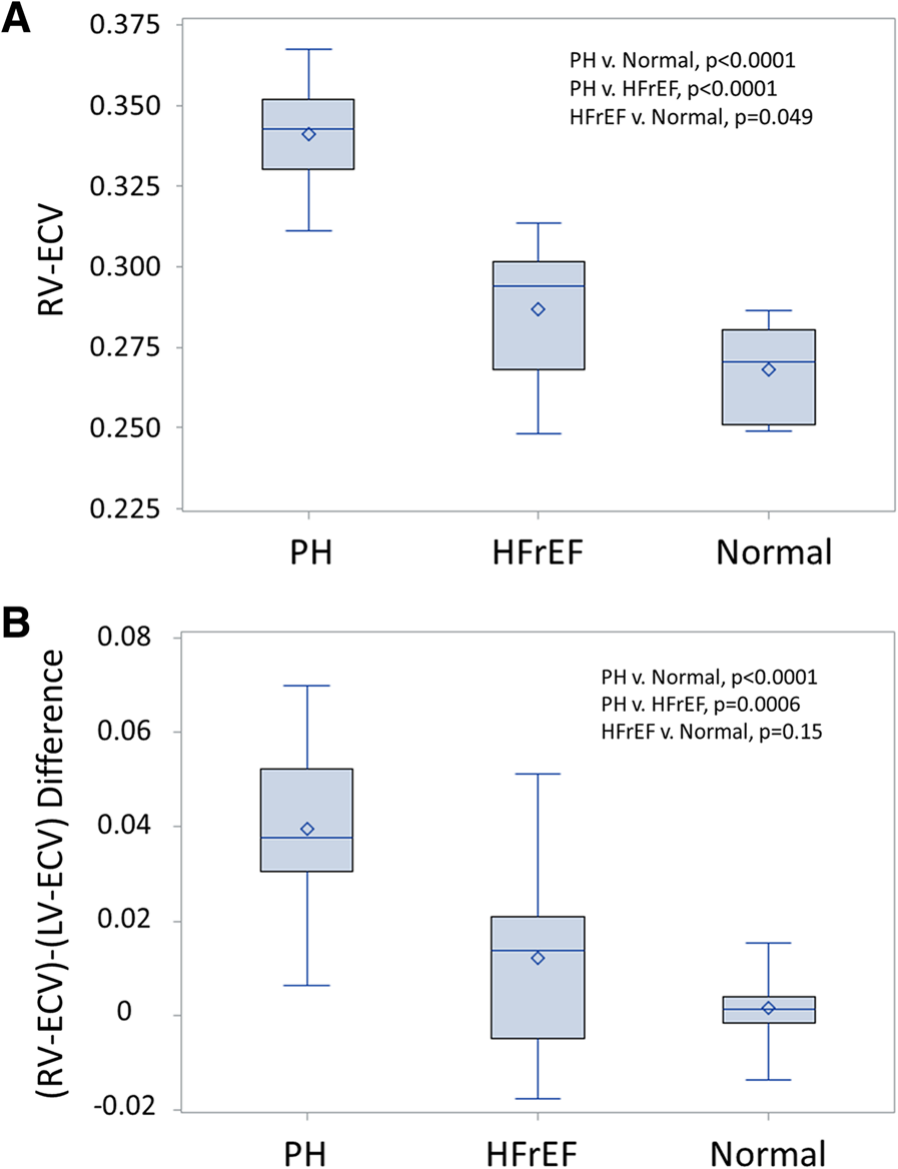
ECV results in PH, HFrEF, and normal volunteers. **a** Box plots are shown for these three groups of patients. RV-ECV is higher in PH versus v HFrEF (*p* < 0.0001), in PH versus normal volunteers (*p* < 0.0001), and in HFrEF versus normal volunteers (*p* = 0.049). **b** Box plots are shown for the differences in RV-ECV and LV-ECV in these three groups of patients. The difference between RV-ECV and LV-ECV is greater in PH versus HFrEF (*p* < 0.0006) and greater in PH versus normal volunteers (*p* < 0.0001). The RV-LV ECV difference in HFrEF versus normal volunteers did not meet statistical significance (*p* = 0.15)

Figure 4a summarizes the ranges for RV-ECV among patients with PH, HFrEF, and normal volunteers. The RV-ECV in PH patients was 27.2 % greater than the RV-ECV in normal volunteers (0.341 v. 0.268; *p* < 0.0001) and 18.9 % greater than the RV-ECV in HFrEF patients (0.341 v. 0.287; *p* < 0.0001). RV-ECV was greater than LV-ECV in PH (RV-LV difference = 0.04), but RV-ECV was nearly equivalent to LV-ECV in normal volunteers (RV-LV difference = 0.002) (*p* < 0.0001 for RV-LV difference in PH versus normal volunteers).

### Independence of RV-ECV from RVEF and detection of occult RV fibrosis

**Fig. 5:**
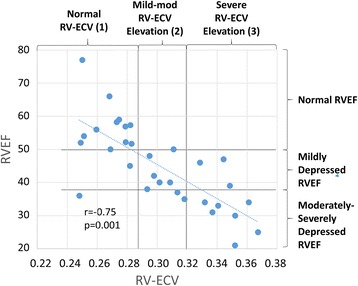
Relationship between RV systolic dysfunction and RV-ECV. Right ventricular ejection fraction (RVEF) and RV extracellular volume fraction (RV-ECV) are each divided into three groups, and the distribution of RV-ECV for different RVEF groups is shown. There is a significant correlation between RVEF and RV-ECV (*r* = −0.75; *p* = 0.001)

In all study participants from all groups, greater RV-ECV by ANGIE was associated with decreasing RVEF by CMR (*r* = −0.75, *p* = 0.001). In Fig. 5, patients were grouped into three RVEF groups and three RV-ECV groups. A normal RV-ECV was defined as less than 28.7 % based on the RV-ECV value at the 99th percentile for RV-ECV in the 10 normal volunteers (range 0.249–0.287). Normal LV-ECV values have also been published for patients with T1 mapping in 1.5 T and 3.0 T magnets [26, 27]; however, we used data from our normal volunteers for the normal RV-ECV values, which are not well-established in the literature. Among the 22 patients in the PH and HFrEF groups, forty-three percent of patients with a normal RVEF had an abnormal RV-ECV (ECV Group 2), indicating a significant prevalence of occult fibrosis despite a normal RVEF. In patients with mildly depressed RVEF, 50 % of the patients had an elevated RV-ECV (ECV Group 2). In patients with a moderately-severely depressed RVEF, there was a range of abnormal ECV, with 43 % having a modest increase in RV-ECV (ECV Group 2), and 57 % having a more severe increase in RV-ECV. These ranges demonstrate that patients with similar degrees of RV dysfunction by RVEF can have variable RV-ECV values (ECV group 3). In fact, RV-ECV ranged from 0.25−0.37 in patients with an abnormal RVEF.

### Multivariable model: RV-ECV, RVEF, and RV end-diastolic volume

**Fig. 6:**
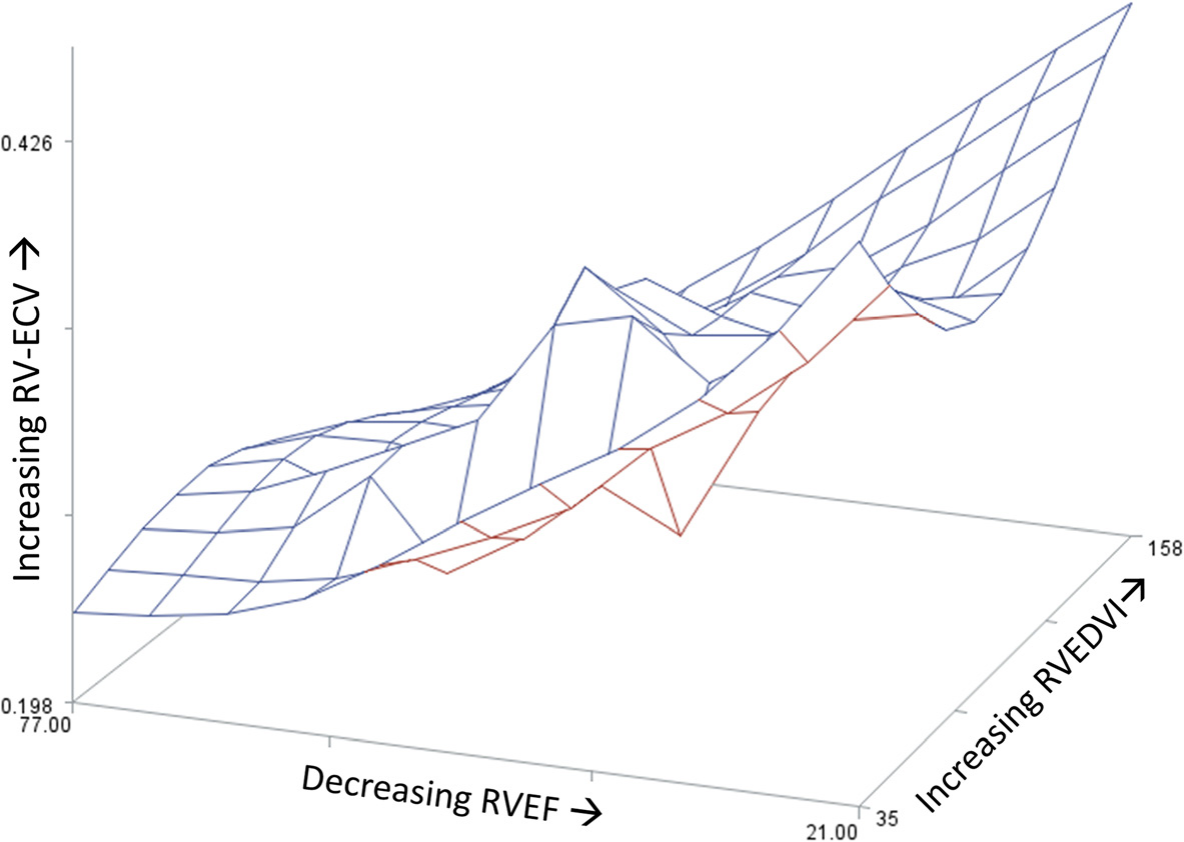
3D depiction of relationships among RV Fibrosis, dysfunction, and dilitation. The 3D surface plot shows the complex relationships among RV-ECV, RVEF, and RVEDVI. Please also refer to Table 3, which shows that patient group, RVEDVI, and RVEF are independently associated with RV-ECV. The surface is highest in the corner corresponding to decreased RVEF and increased RVEDVI

Figure 6 summarizes the complex relationship between RV-ECV, RVEDVI, and RVEF. The multivariable linear model with these variables is described in more detail in Table 3. Both decreasing RVEF and increasing RVEDVI were independently associated with increasing RV-ECV in these patients. Even after adjustment for RVEF and RVEDVI, the clinical diagnostic group (PH v. HFrEF v. Normal) was still independently associated with RV-ECV. The overall R^2^ for this model was 0.804 (adjusted R^2^ = 0.783; *p* < 0.0001). The independent association of RV-ECV with the diagnostic group even after adjustment for RVEF and RVEDVI suggests the presence of independent factors associated with PH relative to other groups that lead to increased ECV independent of the degree of RV dilitation or dysfunction.

**Table 3:** Multivariable model for RV-ECV

Variable	DF	Parameter estimate	Standard error	t value	*p* value
Intercept	1	0.361	0.0182	19.8	<.0001
RVEF	1	−0.000829	0.000360	−2.3	0.03
RVEDVI	1	0.000296	0.000128	2.32	0.03
Diagnostic group	1	−0.0234	0.00563	−4.16	0.0003

### Interobserver variability

We also assessed interobserver variability for RV-ECV and RV T1 values before and after contrast in 10 randomly selected participants with contrast-enhanced MRIs in the PH and HFrEF groups. We found that the ICC for RV-ECV was 0.851, the ICC for the RV pre-contrast T1 value was 0.945, and the ICC for the RV post-contrast T1 value was 0.798.

## Discussion

This is the first report of T1 and ECV determination for the RV in a clinical population of patients with PH or HFrEF without PH. There were several key clinical findings of particular importance to the field of CMR, T1 mapping, RV imaging, ECV determination, and PH. First, ANGIE ECV assessment for the RV is feasible in these patient groups and generates high- resolution results for both the RV and the LV. Second, ANGIE measurements of LV-ECV correlated well with MOLLI measurements of LV-ECV. Although MOLLI measurements of RV T1 values were of insufficient resolution to permit comparison with the higher resolution ANGIE T1 and ECV measurements, the strong correlation between MOLLI and ANGIE for LV-ECV is very important because it shows for the first time that ECV values of the LV as measured with ANGIE are comparable to those determined with MOLLI imaging.

The third significant finding is that ANGIE detected significantly higher RV-ECV values in PH patients compared with normal volunteers and patients with HFrEF without PH. Of note, although RV involvement in HFrEF is not uncommon, particularly those with PH, the patients we chose for the HFrEF cohort did not have PH. In addition, the measures of RV size and function were closer to those of normal volunteers in patients with HFrEF compared with the corresponding measures in patients in the PH group. In particular, RVEDVI was reduced by more than 30 % in HFrEF, while RVESVI was reduced by more than 40 % in HFrEF compared with PH.

A fourth significant finding is that RV-ECV represents a unique parameter describing RV structure that is associated with RVEF and RVEDVI but is not merely a surrogate for one of these parameters. This was shown by the results of the multivariable linear model with RV-ECV as the outcome variable. In this model, RV-ECV was independently associated with both RVEF and RVEDVI modeled as independent variables. Furthermore, the clinical diagnostic group was also highly associated with RV-ECV even after adjustment for RVEF and RVEDVI. This is consistent with independent biologic factors in PH that result in greater RV-ECV in PH compared with patients having HFrEF and normal volunteers, independent of the degree of RV dysfunction and dilitation.

### Imaging of fibrosis and scar in the RV in pulmonary hypertension

The findings justify further study regarding the use of this RV fibrosis imaging technique in patients with PH [3–6]. While noninvasive imaging methods have shown several measurable indices to be reliable surrogates for RV function (such as RV strain [11]), there has not been an effective way to image diffuse RV fibrosis prior to the present study. While the use of LGE can be used to localize focal fibrosis [12, 28], its use in patients with PH has been limited to imaging of LV septal fibrosis at the RV insertion points [29, 30]. Although it is clear that fibrosis is important in the RV in PH, traditional imaging of scar with LGE in the RV free wall is technically difficult. Furthermore, while T1 mapping protocols such as MOLLI have been used to quantify the myocardial ECV in the LV, their use in the RV has been limited because of inadequate spatial resolution.

### ANGIE as a solution to the inherent challenges of RV T1 mapping

ANGIE presents a unique solution to T1 mapping of the thin RV free wall. Just as we showed the high quality of native ANGIE imaging of the RV in a recent publication [20], the high quality of post-contrast ANGIE T1 mapping of the RV is shown in Fig. 1. This high resolution is achieved by several innovative imaging techniques, described previously. Briefly, ANGIE employs navigator gating to allow acquisition of images during free breathing and, consequently, to achieve higher spatial resolution. To do so in a reasonable scan time, ANGIE also uses compressed sensing and parallel imaging for acceleration. These innovations provide high resolution imaging of the thin-walled RV in a very acceptable period of imaging time. ANGIE results for both the RV and LV can be obtained during a single study and using the same contrast injection used for LGE imaging of the LV, also performed during the study. As described in the methods, one pre-contrast native ANGIE acquisition and two post-LGE ANGIE acquisitions are typically performed. Analysis of RV-ECV was found to be very reproducible. The ICC of 0.945 for interobserver agreement for pre-contrast RV T1 values was also quite high and consistent with values obtained in volunteers in our previously published study using this technique [20]. The ICC for post-contrast RV T1 values was also very good but slightly lower than that for the RV pre-contrast T1 values.

### Clinical applications and significance

There are a number of potential clinical applications of RV fibrosis assessment in patients with PH and others with HFrEF. First, RV-ECV could be used for risk stratification of patients with PH in combination with RVEF, if additional follow-up data confirm that RV-ECV, like RVEF, is associated with adverse clinical outcomes. Second, RV-ECV assessment could be used to monitor the effectiveness of various therapies [31–33] for PH by performing longitudinal CMR assessments with RV-ECV measurements. Third, RV-ECV assessment could be used in animal models of PH in the context of drug development to compare how novel therapeutic agents modify the effect of PH on RV fibrosis. Fourth, RV-ECV mapping could be used to evaluate RV reserve in patients being evaluated for cardiac surgery, pulmonary endartectomy, or lung transplantation, as early postoperative right ventricular failure is a significant cause of morbidity and mortality in these patients.

### Limitations

While the sample size of the present study is adequate to assess the feasibility and high quality of this method for RV-ECV determination, as well associations between RV-ECV and other patient findings, future studies with ANGIE in PH to evaluate associations with clinical events are indicated but would require a larger number of patients. We also acknowledge that fat suppression could be a potential contaminant of RV T1 and ECV measurements. Of note, we used fat suppression for the purpose of limiting partial volume contamination by fat; however, ANGIE images can be acquired without fat suppression. Although MRI ECV measurements have been found to correlate with histologic fibrosis, and we interpreted elevated ECV in this study as a surrogate for fibrosis, inflammation and edema may also contribute to an increased ECV. There were differences in gender in the groups, but both the PH and volunteer group were similar in the sense that both had more females than males. Volunteers were younger than PH and HFrEF patients, but it has recently been demonstrated that age does not have a significant effect on CMR measurements of ECV [34]. Another limitation is that ANGIE was applied to only two slices. To overcome the latter limitation, we are developing 3D ANGIE methods for whole heart coverage and more efficient RV imaging in the future [35].

## Conclusions

Pre- and post-contrast ANGIE imaging provides high-resolution T1 mapping and ECV assessments for not only the LV but also the RV, with LV-ECV by ANGIE and MOLLI in close agreement. Elevated RV-ECV in PH is independently associated not only with RV chamber dilitation and dysfunction, but also with the clinical diagnostic group, suggesting that increased RV-ECV/RV fibrosis in PH results from biologic factors that cannot be predicted by volumetric RV findings on CMR. This highlights the importance of assessing RV-ECV in addition to RV volumes and EF in PH and other conditions that may adversely affect the RV. Further studies of RV-ECV in PH and HFrEF are indicated to assess associations among RV-ECV, functional limitations, and long-term clinical outcomes, as well as the effects of drug therapy for PH on RV-ECV.

## Abbreviations

ANGIE: Accelerated and navigator-gated Look-Locker imaging for cardiac T1 estimation
CMR: Cardiovascular magnetic resonance
CTEPH: Chronic thromboembolic pulmonary hypertension
DTPA: Diethylene triamine pentaacetic acid
ERA: Endothelin receptor antagonists
FOV: Field of view
HFrEF: Heart failure with reduced ejection fraction
λ-Gd: Partition coefficient of gadolinium
LGE: Late gadolinium enhancement
LV-ECV: Left ventricular extracellular volume fraction
LVEDVI: Left ventricular end-diastolic volume index
LVEF: Left ventricular ejection fraction
LVESVI: Left ventricular end-systolic volume index
MOLLI: Modified Look-Locker inversion recovery
PH: Pulmonary hypertension
RV-ECV: Right ventricular extracellular volume fraction
RVEDVI: Right ventricular end-diastolic volume index
RVEF: Right ventricular ejection fraction
RVESVI: Right ventricular end-systolic volume index
TE: Echo time
TR: Repetition time
WHO: World Health Organization
